# Pre-clinical animal models are poor predictors of human toxicities in phase 1 oncology clinical trials

**DOI:** 10.1038/s41416-020-01033-x

**Published:** 2020-09-01

**Authors:** Johnique T. Atkins, Goldy C. George, Kenneth Hess, Kathrina L. Marcelo-Lewis, Ying Yuan, Gautam Borthakur, Sean Khozin, Patricia LoRusso, David S. Hong

**Affiliations:** 1 DeBakey Heart and Vascular Center, Houston Methodist Research Center, Houston, TX USA; 2 Department of Symptom Research, The University of Texas MD Anderson Cancer Center, Houston, TX USA; 3 Department of Biostatistics, The University of Texas MD Anderson Cancer Center, Houston, TX USA; 4 The University of Texas MD Anderson Cancer Center, Houston, TX USA; 5 Department of Leukemia, The University of Texas MD Anderson Cancer Center, Houston, TX USA; 6 United States Food and Drug Administration, Rockville, MD USA; 7 Yale Cancer Center, New Haven, CT USA; 8 Department of Investigational Cancer Therapeutics, The University of Texas MD Anderson Cancer Center, Houston, TX USA

**Keywords:** Comorbidities, Oncology

## Abstract

**Background:**

Our objective was to determine the correlation between preclinical toxicity found in animal models (mouse, rat, dog and monkey) and clinical toxicity reported in patients participating in Phase 1 oncology clinical trials.

**Methods:**

We obtained from two major early-Phase clinical trial centres, preclinical toxicities from investigational brochures and clinical toxicities from published Phase 1 trials for 108 drugs, including small molecules, biologics and conjugates. Toxicities were categorised according to Common Terminology Criteria for Adverse Events version 4.0. Human toxicities were also categorised based on their reported clinical grade (severity). Positive predictive values (PPV) and negative predictive values (NPV) were calculated to determine the probability that clinical studies would/would not show a particular toxicity category given that it was seen in preclinical toxicology analysis. Statistical analyses also included kappa statistics, and Matthews (MCC) and Spearman correlation coefficients.

**Results:**

Overall, animal toxicity did not show strong correlation with human toxicity, with a median PPV of 0.65 and NPV of 0.50. Similar results were obtained based on kappa statistics and MCC.

**Conclusions:**

There is an urgent need to assess more novel approaches to the type and conduct of preclinical toxicity studies in an effort to provide better predictive value for human investigation.

## Background

The goal of preclinical studies in drug development is to predict the behaviours of therapeutic agents in humans. Efficacy, toxicity, and dosing requirements are important factors for the success of these agents, and pharmaceutical companies invest many resources to determine which agents are most likely to be successful. Combinations of in vitro and in vivo studies are conducted according to experience, historical precedence, and governmental requirements, but there is no consensus about the actual predictability of these preclinical studies. Drugs that show efficacy in cell model systems are not always beneficial clinically. Additionally, it is very difficult to determine toxicity from these in vitro models. The predictability of in vivo models of drug toxicity is also unclear. Historically, it appears unrealistic to expect preclinical models to predict the exact behaviour of drugs in humans.

Cytotoxic anti-cancer agents, by nature, tend to have high toxicity, especially when given to patients with already compromised health. Oncology drugs currently in development are highly diverse, including targeted agents aimed at specific genetic mutations or overactive tumorigenesis and metastasis pathways. These agents include small molecules, antibodies, anti-sense, and conjugates and can have toxicities far different from those of traditional cytotoxic anti-cancer agents. The correlation between outcomes of preclinical models and results seen in early-Phase clinical trials is not well studied. In the current study, we sought to determine the ability of preclinical animal models to predict toxicities seen in Phase 1 clinical trials of oncology drugs. Our goal was to determine through a meta-analysis whether the current method of conducting preclinical analysis of toxicities in animals accurately predicts the toxicities of the new wave of targeted oncology drugs.

## Methods

### Data collection

We obtained a convenience sample of 120 investigational brochures of drugs that were assessed between 2005 and 2013 in single-agent Phase 1 clinical trials. Phase 1 toxicities were published for 108 of these 120 drugs. Among these 108 drugs included in our analysis, 97 were assessed in Phase 1 clinical trials conducted at The University of Texas MD Anderson Cancer Center in Houston, Texas: 87 in the Phase 1 clinical program and 10 in the Department of Leukaemia. The remaining 11 drugs were assessed in Phase 1 clinical trials conducted at the Karmanos Cancer Institute in Detroit, Michigan.

Toxicities were categorised according to Common Terminology Criteria for Adverse Events version 4.0. These categories are listed in Table 1, along with the most common toxicities included in each category. For both preclinical and clinical data, toxicities were assessed according to the categories and not the specific toxicities (e.g., either a seizure or a headache would indicate that a drug has neurologic/psychiatric toxicities). A drug was considered to have a particular human toxicity if the Phase 1 study mentioned that the toxic effect occurred in at least one patient. A drug was considered to have a particular animal toxicity if that toxic effect was observed in at least one animal in that particular animal model. If a particular toxicity was not mentioned in the Phase 1 report, it was assumed to have not been observed. Only toxicities that were attributed to the study drug were recorded. Data were collected by J.T.A. and independently verified. If a toxicity category was not assessed in a preclinical animal model, that category was omitted for comparison with human toxicities. For clinical data, grade 3 or 4 toxicities were noted during data collection.

**Table 1:** Toxicity categories used in the phase I study reports examined, as per the Common Terminology Criteria for Adverse Events, version 4.0.

Toxicity category	Common human toxicities	Common animal toxicities
Cardiovascular	Hypertension, tachycardia, bradycardia, dyslipidemia	Tachycardia, bradycardia
Cutaneous	Rash, alopecia	Rash, fur discoloration
Endocrine	Diabetes	Increase in endocrine system organs
Gastrointestinal	Nausea, vomiting, diarrhoea	Loose stool
General	Fatigue, fever, chills	Decreased activity
Haematologic	Neutropenia, thrombocytopenia	Changes in red blood cells or white blood cells
Hepatic	Changes in alanine transaminase or aspartate transaminase	Changes in alanine transaminase or aspartate transaminase
Metabolic	Anorexia, decreased appetite, hyperglycaemia	Decreased food consumption
Musculoskeletal	Muscle pain, back pain	Awkward gait, hunched posture
Neurologic/psychiatric	Headache, insomnia	Tremors
Ocular	Blurred vision	Bloody tears
Renal	Changes in creatinine or blood urea nitrogen	Blood in urine
Respiratory	Cough, shortness of breath	Laboured breathing

### Statistical analysis

Positive predictive values (PPV) and negative predictive values (NPV) were used to determine the probability that human Phase 1 studies would or would not show a particular category of toxicity given that it was seen in preclinical toxicology analysis. Thus, PPV represents the probability that human Phase 1 studies will show a particular toxicity given that animal data show that toxicity. NPV is the probability that human Phase 1 studies will not show a particular toxicity given that animal data do not show that toxicity. Kappa statistics, balanced accuracy rate (BAR), and Matthew’s correlation coefficient (MCC) were also computed to validate the results: kappa provides a measure of the degree of concordance between clinical and preclinical toxicity data, BAR is the average of sensitivity and specificity, and MCC was used to estimate the predictive accuracy of preclinical data for adverse events in humans.

## Results

### Drug and data characteristics

The 108 oncology drugs included in this study included 90 small molecules (83%), 15 biologics (14%), and three conjugates (3%). Definitions of these drug classes are included here for the reader’s benefit. Small molecules are drugs of usually a chemical origin and of low molecular weight that can enter cells easily, and then impact downstream molecules/proteins within the cell.^1^ Examples of small molecule drugs included in molecularly targeted therapy include protein kinase antagonists, such as tyrosine kinase inhibitors. Biologics are generally large and complex molecules that may be produced utilising biotechnology in a living biological system, such as a microorganism, plant cell or animal cell.^2^ Examples of biologics included in early-Phase clinical trials include monoclonal antibodies, vaccines and recombinant proteins. Antibody-drug conjugates are highly specific immune-conjugates, consisting of a monoclonal antibody (targeted to a specific tumour cell surface antigen) joined via a chemical linker to a powerful cytotoxic anti-cancer drug (referred to as a cytotoxic payload).^3^ The antibody-drug conjugate facilitates delivery of an ultra-toxic payload directly to targeted cancer cells.^3^ Of the 108 drugs included in this study, 90 drugs (83%) were targeted therapies and 18 (17%) were considered traditional chemotherapy.

We assessed the 13 categories of toxicities in four animal models, for a total of 52 conditions. The data available for each toxicity category and animal model varied for each drug. Over the 52 conditions, the median number of drugs with data was 57.5 (the minimum was 24 and the maximum was 90). A total of 90 drugs (83% of drugs) had data available for rats, 77 (71%) for dogs, 40 (37%) for monkeys and 28 (26%) for mice.

### Toxicity characteristics

**Fig. 1:**
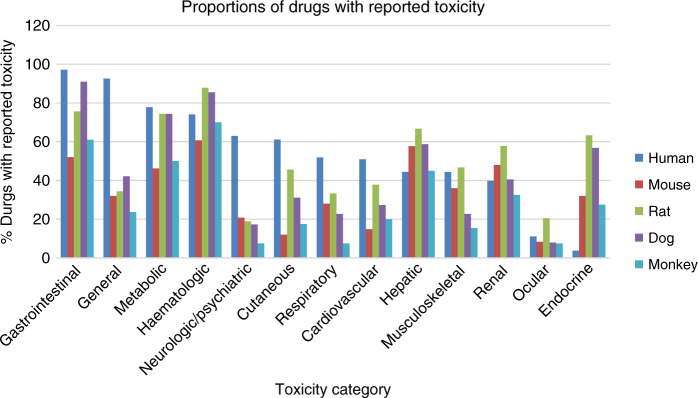
Bar charts show proportion of drugs with reported toxicity in humans and in each animal model. Blue bars: humans; red: mouse; green: rat; purple: dog, light blue: monkey.

The rat was the most common animal model used to assess preclinical toxicities; of 108 drugs considered, 90 drugs (83%) had at least one toxicity category assessed using this model. Of 108 drugs, 77 drugs (71%) had at least 1 toxicity reported in dogs, 40 (37%) in monkeys, and 28 (26%) in mice. The proportion of drugs with reported toxicity in humans and in each animal model is shown in Fig. 1. In humans, the most commonly reported toxicity categories for all grades were gastrointestinal (97% of drugs) and general (93%). The least commonly reported toxicity categories for all grades in humans were endocrine (4%) and ocular (11%). For grades 3 and 4, the most commonly reported categories were gastrointestinal (69%) and haematologic (62%). Haematologic toxicities were the most commonly reported category in rats (88%), monkeys (70%), and mice (61%) and the second most commonly reported in dogs (86%). Gastrointestinal toxicities were the most commonly reported category in dogs (91%) and the second most commonly reported in rats (76%) and monkeys (61%). Ocular and neurologic/psychiatric toxicities were the least commonly reported category in rats, dogs, and monkeys. Toxicities not captured well in preclinical studies relative to human studies included neurologic/psychiatric, cutaneous, respiratory, and cardiovascular toxicities.

### Agreement between preclinical and clinical toxicities

**Fig. 2:**
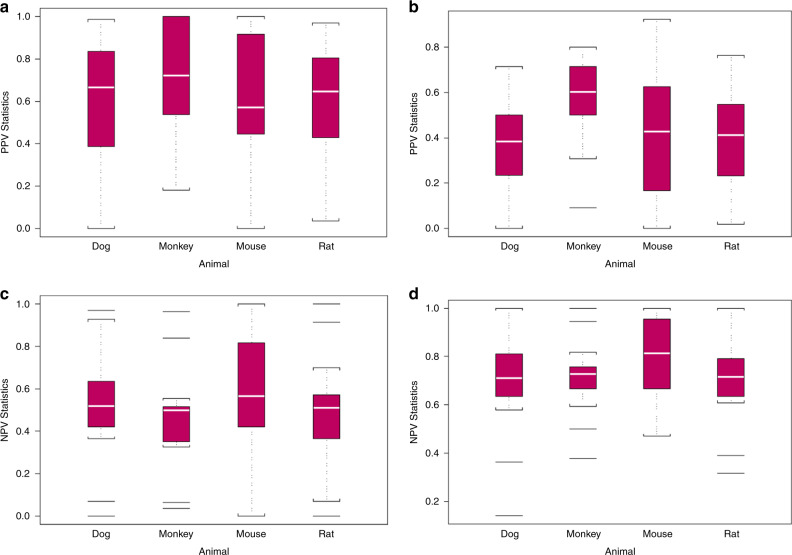
Box and whisker plots show quartiles of the distribution of positive predictive values (PPVs) and negative predictive values (NPV) of human toxicities based on animal toxicities. For each animal model, **a** PPV for all toxicity grades (median: dog = 0.67, monkey = 0.72, mouse = 0.57, rat = 0.65), **b** PPV for grade 3 and 4 toxicities (median: dog = 0.38, monkey = 0.60, mouse = 0.43, rat = 0.41), **c** NPV for all toxicity grades (median: dog = 0.52, monkey = 0.50, mouse = 0.57, rat = 0.51), and **d** NPV for grade 3 and 4 toxicities (median: dog = 0.71, monkey = 0.73, mouse = 0.81, rat = 0.72).

**Fig. 3:**
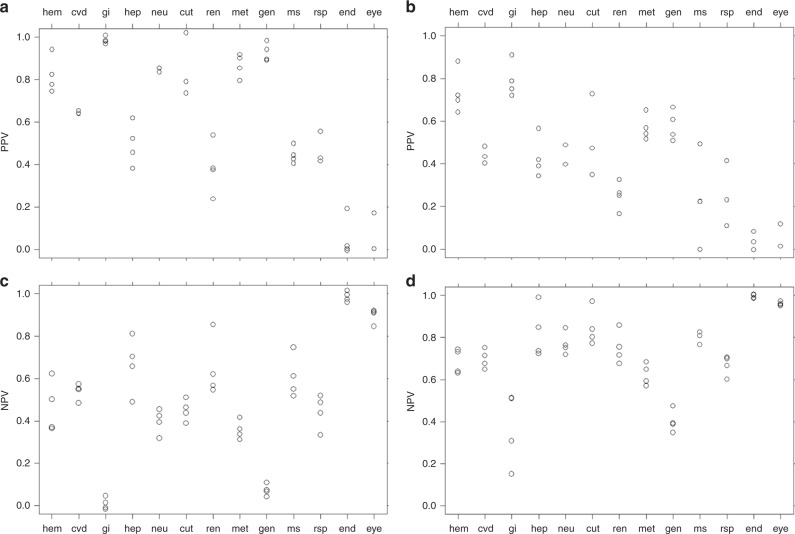
PPV and NPV show the likelihood of each category of toxicity as seen in humans. **a** PPV for all toxicity grades, **b** PPV for grade 3 and 4 toxicities, **c** NPV for all toxicity grades, and **d** NPV for grade 3 and 4 toxicities. The circles in this figure represent the different animal model systems. Abbreviations: hem, haematologic; cvd, cardiovascular; gi, gastrointestinal; hep, hepatic; neu, neurologic/psychiatric; cut, cutaneous; ren, renal; met, metabolic; gen, general; ms, musculoskeletal; rsp, respiratory; end, endocrine; eye, ocular.

All four animal models showed broadly similar PPVs, however the animal model of the monkey had the highest median PPV for all grade toxicities as well as for grade 3 and 4 toxicities in humans (Fig. 2a, b). As with PPV, all four animal models showed broadly similar NPVs (Fig. 2c, d). For all toxicity grades, the highest PPVs were for gastrointestinal, general, and metabolic toxicities and the lowest PPVs were for endocrine and ocular toxicities (Fig. 3a). For grades 3 and 4, the highest PPVs were for haematologic and gastrointestinal toxicities and the lowest were for endocrine and ocular toxicities (Fig. 3b). For all toxicity grades and for grades 3 and 4, the highest NPVs were for endocrine and ocular toxicities and the lowest NPVs were for gastrointestinal and general toxicities (Fig. 3c, d).

For grades 3 and 4, there were no conditions in which PPV and NPV were both >0.75, but there were six conditions for which PPV and NPV were both >0.6. These included haematologic toxicities in all four animal models, as well as cutaneous and metabolic toxicities in monkeys. For all grades, haematologic toxicity in mice was the only condition in which PPV and NPV were both >0.6. Results of the analyses for kappa and MCC are included in the supplement (Supplementary Figs. S1 and S2 and Supplementary Tables S1 and S2).

## Discussion

The primary aim of our study was to evaluate the ability of preclinical animal models (rodent and non-rodent) to predict the toxicity profiles of novel oncology drugs in Phase 1 clinical trials. Our comparison of preclinical and clinical toxicity profiles of 108 oncology drugs showed that animal models did not accurately predict the toxicity profile of the drugs in humans. There can be several explanations for the lack of correlation between preclinical and phase I clinical toxicity profiles seen in the current study. First, Phase 1 clinical trials have a relatively small number of patients, and many toxicities are experienced only by a few patients. Data from late-Phase studies may provide a more accurate assessment of the true toxicities of a particular drug. Second, there are documented flaws in the collection, assessment and reporting of patient toxicity data,^4–6^ as well as difficulties in assessing certain toxicity categories in animals, and these flaws may have contributed to the lack of correlation. Third, investigational brochures represent abbreviations or summaries of preclinical and clinical data, and thus do not contain all the data in the good laboratory practice (GLP) toxicity studies. It is possible, though quite unlikely, that certain human toxicities were mentioned in the GLP toxicity studies but had not been included in the investigational brochures.

Several older, smaller studies of anti-cancer drugs showed qualitatively similar toxicities in animals and humans; dog models predicted gastrointestinal toxicities particularly well, and data from dogs and monkeys over-predicted hepatic and renal toxicities in humans.^7–9^ From these studies, Rozencweig et al.^8^ concluded that the predictability of animal data is highly dependent on the prevalence of the particular human toxicity, and a few toxicities are virtually unpredictable by animal data. Furthermore, these authors concluded that preclinical investigation of organ system toxicity in animals may not be useful for the experienced clinician who is already knowledgeable of the common toxicities of chemotherapy drugs in early-Phase clinical trials. The current study includes newer data that account for the fact that current early-Phase oncology clinical trials include novel therapies, such as molecularly targeted therapy.

In another study of 150 drugs for many different therapeutic indications, including anti-cancer drugs, Olson et al.^10^ determined that preclinical toxicology studies were valuable in predicting significant human toxicities and in identifying categories of human toxicities. In that study, the authors used toxicity categories similar to those used in the Common Terminology Criteria for Adverse Events, as we did in the current study. Their analysis showed that a combination of rodent and non-rodent studies had a positive concordance for values in humans for 71% of drugs, and concordance was 63% for non-rodent studies alone and 43% for rodent studies alone. The highest overall concordance was seen in haematologic, gastrointestinal, and cardiovascular toxicities, and the lowest concordance was seen in cutaneous toxicities.

An example of a high-profile case where acute toxicity prediction went wrong was that seen with TGN1412^11^ where cytokine storm and multi-organ failure were observed in humans but not to a similar extent in preclinical studies. This changed practice of staggering patients in early-Phase studies and the present analysis further supports this practice. Given the challenges in properly attributing toxicities to oncology treatments, as well as the major effect these toxicities can have on patients who may already have a high burden of disease, it would be beneficial to be able to accurately assess potential toxicities in a preclinical setting. However, there are several sources of uncertainty in animal toxicity tests that affect extrapolation to humans, including but not limited to the following: (1) species, strain, and sex variations; (2) scaling of doses appropriate for small, short-lived animals (usually rodents) to larger doses for large, long-lived animals (humans); (3) variability of dosing routes; and (4) homogeneity (genetic and otherwise) of most test animal populations relative to human patient populations.^12^ Although the importance of these factors has been acknowledged, it has been argued that animal models can still be useful predictors of human toxicities provided that these factors are considered appropriately. Another important aspect to consider is that drugs with significant preclinical toxicities are not likely to advance to the clinic and therefore could not be included in this analysis. This leaves us to analyse drugs that behaved relatively well in preclinical studies, but no comparison to those that did not. Similarly, potentially serious toxicities identified in animal models are often managed by instituting mitigation procedures, and therefore lack of concordance in some cases may be a demonstration of success rather than failure.

The FDA and other regulatory agencies are starting to require drug companies to provide more data to support the selection of specific species (and even strains) to test new drugs. A given animal model may be deemed inappropriate if that animal lacks an appropriate drug target, has an irrelevant target, or metabolises the drug differently from humans. During our data collection, we encountered several instances in which an animal model was excluded from toxicity testing owing to the aforementioned reasons. However, most of the investigational brochures did not provide an explanation for the selection of animal models, leading us to believe that if there was not a reason to exclude a particular model, no reason was needed to include or justify the use of a particular model.

We made considerable effort to collect data that would enable a direct comparison between animal and human toxicities but recognised at the study onset that the data could not completely answer the question at hand. For that to be possible, each toxicity would need to be evaluated and reported in both preclinical and clinical studies, which is not always the case. For example, several small molecules have skin rash as a toxicity, and this is often underreported in animal toxicity studies. In addition, toxicities are often evaluated differently in animals and humans. In animals, individual organs are removed and assessed for physiological changes, whereas in humans, laboratory changes in blood and urine are relied upon to assess these changes. Also, toxicities that can be vocalised by humans, such as fatigue, pain, and dizziness, have to be assessed by observation in animals.

Considering the limitations of comparing toxicity profiles of animals and humans, our analysis has some strengths. In addition to PPV and NPV analyses, kappa, MCC, and BAR statistical analyses were performed and are presented in the supplement, and these were in agreement with each other in determining the lack of correlation. In addition, our results are in agreement with previous reports showing that haematologic toxicities are the most likely to be predicted for humans by animal models.^8,10,13^ The objective nature of haematologic data, as opposed to observational data, could explain this phenomenon.

The lack of correlation in terms of efficacy between animal models and humans has been well documented and illustrates how different species can produce different drug effects. Many drug candidates are moved into clinical testing on the basis of preclinical data showing efficacy for a desired indication in animals, only for the drug to be deemed ineffective in patients. In our analysis, we were limited by the number of drugs with sufficient preclinical and clinical data available. Although 108 drugs were enough to reliably estimate measures of agreement and predictive capacity in our analysis, a larger study encompassing more oncology drugs would provide more definitive conclusions, and we have planned such a study using an FDA database of toxicity profiles.

In light of the current analysis, given the paucity of agents advancing into the clinic (or the need for variation before advancement), there is an urgent need to assess more novel approaches to preclinical toxicity studies to provide better predictive value for human investigation. Balas and Ellis^14^ proposed a three-tier principle of transparency, replication, and triangulation that should be achieved before publication in *Nature*, to ensure that the results warrant further study in preclinical and clinical trials. Everitt^15^ introduced a similar concept of the three R’s—relevance, robustness, and reproducibility—in an effort to enhance the translational value of preclinical animal efficacy studies. The current focus of the National Institutes of Health, including the National Cancer Institute, is for investigators to provide rigor and transparency when conducting and reporting animal studies. Although the industry and FDA scientists agree that animal studies are generally not reproducible or reliable, general agreement about what should be done to ensure better use of these models is lacking.^16^ More accurate, detailed, and standardised assessment of toxicities in preclinical models and in clinical trials in humans may be critical in improving the drug safety of novel oncologic agents.

## Supplementary information

Supplementary Material

## Data Availability

Data are available from D.S.H. with a reasonable request.
