# Supplementary material for: Pre-clinical animal models are poor predictors of human toxicities in phase 1 oncology clinical trials
Source: Br J Cancer. 2020 Sep 1;123(10):1496–501. doi: 10.1038/s41416-020-01033-x (PMC7652903; doi:10.1038/s41416-020-01033-x)
Supplement: Supplementary file 1 — Supplementary Material [file 41416_2020_1033_MOESM1_ESM.docx]

**Supplementary methods**

The proportion of drugs attributed to each category of toxicity was computed for both the preclinical and the clinical data. Kappa statistics were calculated to assess agreement between toxicities in animal models and in humans, for all toxicity grades and for grades 3 and 4 only, for each type of animal (mouse, rat, dog, or monkey), and for each category of toxicity. The interpretation of the kappa values (maximum possible range -1 to +1) was based on Landis and Koch’s classification [Landis JR, Koch GG: The measurement of observer agreement for categorical data. Biometrics. 1977;33:159-174]: kappa values ranging from -1.0 to 0.0 denoted poor agreement; 0.0 to 0.2, slight agreement; 0.21 to 0.4, fair agreement; 0.41 to 0.6, moderate agreement; 0.61 to 0.8, substantial agreement; and 0.81 to 1.0, almost perfect agreement. The Matthews correlation coefficient (MCC) was also computed to assess the predictive accuracy of preclinical data for human toxicities, for all toxicity grades and for grades 3 and 4 only, for each animal, and for each category of toxicity. The Spearman correlation coefficient was calculated and a scatterplot was created to visualize the relationship between kappa values and MCC values for all toxicity grades and for grades 3 and 4 only. We further validated our data by computing the balanced accuracy rate (BAR), which is the average of sensitivity and specificity. Data were not stratified by type (small molecule, biologic, or conjugate) or nature (chemotherapy, targeted) of drug owing to small numbers.

**Supplementary results**

The Spearman’s rank correlation coefficient between kappa values for all grades and those for grades 3 and 4 was 0.64 (Figure S1). With 13 toxicity categories and four animal models, 52 kappa statistics were calculated for all toxicity grades and another 52 for grades 3 and 4 (Table S1). Animal models were not combined for analysis; each model was individually compared with clinical data to calculate kappa statistics. The median kappa value for both was 0.06. For grades 3 and 4, 41 (79%) of the 52 kappa values were <0.2 (indicating poor or slight agreement between humans and animals); for all grades, 44 (85%) of the 52 kappa values were <0.2. Of the 104 kappa values, only four were >0.4 (indicating moderate or substantial agreement between humans and mice only): hematologic toxicities for all grades (κ = 0.61) and hematologic (κ = 0.62), gastrointestinal (κ = 0.43), and cutaneous (κ = 0.62) toxicities for grades 3 and 4.

MCCs were very similar to kappa values (Table S2) and highly correlated with kappa values for grades 3 and 4 (r_S_ = 0.99) and for all grades (r_S_ = 0.96; Figure S2). The r_S_ value between BAR and kappa was 0.91, and between BAR and MCC, 0.95.

**Supplementary figures and tables (online only)**

**Figure S1.** Scatterplot of kappa statistics for all toxicity grades and for grades 3 and 4 in humans.

**Figure S2.** Scatterplots showing the correlation between kappa values and Matthews correlation coefficients for (a) all toxicity grades and (b) grades 3 and 4 in humans and preclinical animal models.

**Figure S1**


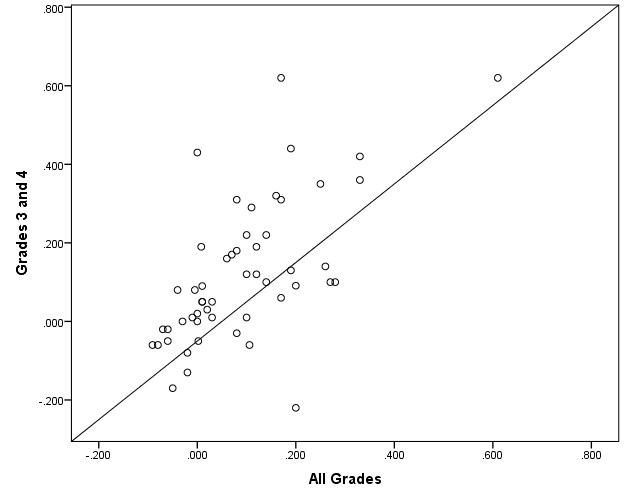


**Figure S2**

**(a)**


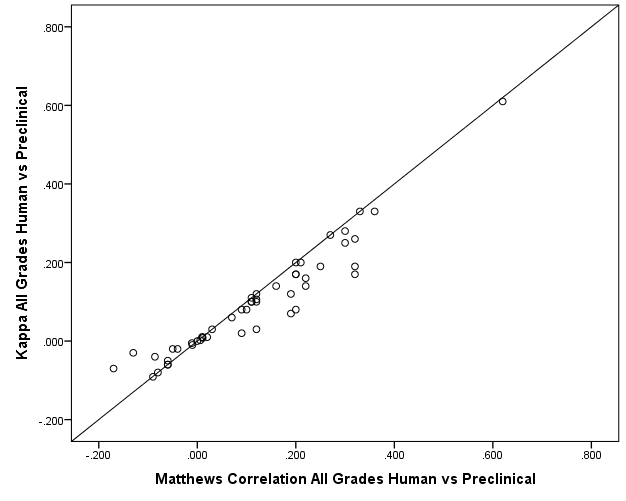


**(b)**


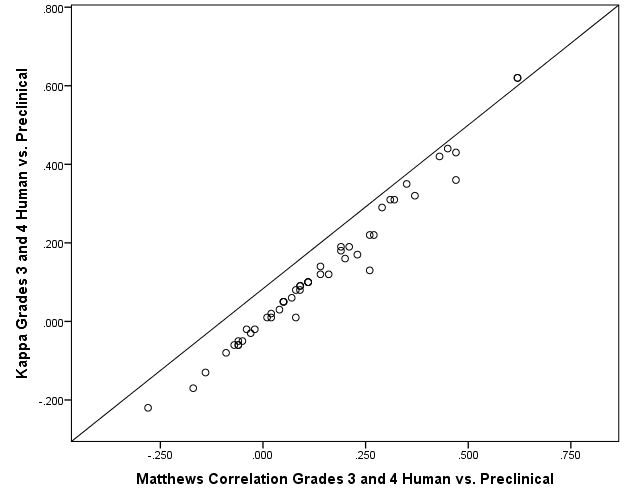


**Table S1.** Kappa agreement between toxicities reported in humans and those reported in animal models

| Toxicity | Mice | | | Rats | | | Dogs | | | Monkeys | | |
| --- | --- | --- | --- | --- | --- | --- | --- | --- | --- | --- | --- | --- |
|  | к | Standard error | No. of drugs | к | Standard error | No. of drugs | к | Standard error | No. of drugs | к | Standard error | No. of drugs |
| All grades |  |  |  |  |  |  |  |  |  |  |  |  |
| Hematologic | 0.61 | 0.15 | 28 | 0.06 | 0.10 | 90 | 0.10 | 0.12 | 76 | 0.33 | 0.16 | 40 |
| Cardiovascular | 0.16 | 0.14 | 27 | 0.20 | 0.10 | 90 | 0.17 | 0.10 | 77 | 0.08 | 0.12 | 40 |
| Gastrointestinal | 0 |  | 25 | -0.04 | 0.03 | 90 | -0.02 | 0.02 | 77 | 0.08 | 0.07 | 41 |
| Hepatic | 0.33 | 0.17 | 26 | 0.14 | 0.09 | 90 | 0.03 | 0.11 | 75 | 0.11 | 0.15 | 40 |
| Neurologic/ psychiatric | 0.01 | 0.15 | 24 | 0.12 | 0.06 | 90 | 0.14 | 0.07 | 75 | 0.07 | 0.04 | 40 |
| Cutaneous | 0.17 | 0.10 | 25 | 0.28 | 0.10 | 90 | 0.17 | 0.10 | 74 | 0.19 | 0.08 | 40 |
| Renal | 0.10 | 0.16 | 25 | -0.06 | 0.10 | 90 | 0.00 | 0.12 | 74 | 0.08 | 0.15 | 40 |
| Metabolic | 0.26 | 0.15 | 26 | 0.12 | 0.11 | 90 | 0.27 | 0.13 | 74 | 0.25 | 0.13 | 40 |
| General | -0.005 | 0.10 | 25 | 0.002 | 0.04 | 90 | -0.02 | 0.06 | 76 | 0.02 | 0.02 | 38 |
| Musculoskeletal | 0.20 | 0.20 | 25 | -0.01 | 0.11 | 90 | 0.01 | 0.11 | 75 | 0.008 | 0.12 | 39 |
| Respiratory | 0.01 | 0.17 | 25 | -0.06 | 0.10 | 90 | -0.05 | 0.10 | 75 | -0.07 | 0.08 | 40 |
| Endocrine | 0 | 0.00 | 25 | 0.03 | 0.02 | 90 | -0.03 | 0.03 | 74 | 0.19 | 0.15 | 40 |
| Ocular | -0.09 | 0.05 | 24 | 0.10 | 0.11 | 87 | -0.08 | 0.02 | 76 | 0.106 | 0.18 | 40 |
| Grades 3 and 4 |  |  |  |  |  |  |  |  |  |  |  |  |
| Hematologic | 0.62 | 0.15 | 28 | 0.16 | 0.09 | 90 | 0.22 | 0.108 | 76 | 0.42 | 0.14 | 40 |
| Cardiovascular | 0.32 | 0.18 | 27 | 0.091 | 0.106 | 90 | 0.06 | 0.11 | 77 | 0.18 | 0.16 | 40 |
| Gastrointestinal | 0.43 | 0.17 | 25 | 0.08 | 0.11 | 90 | -0.08 | 0.08 | 77 | 0.31 | 0.15 | 41 |
| Hepatic | 0.36 | 0.13 | 26 | 0.22 | 0.08 | 90 | 0.05 | 0.10 | 75 | 0.29 | 0.15 | 40 |
| Neurologic/ psychiatric | 0.05 | 0.21 | 24 | 0.19 | 0.11 | 90 | 0.10 | 0.12 | 75 | 0.17 | 0.14 | 40 |
| Cutaneous | 0.62 | 0.25 | 25 | 0.10 | 0.10 | 90 | 0.31 | 0.12 | 74 | 0.44 | 0.16 | 40 |
| Renal | 0.01 | 0.15 | 25 | -0.02 | 0.09 | 90 | 0.02 | 0.11 | 74 | -0.03 | 0.16 | 40 |
| Metabolic | 0.14 | 0.19 | 26 | 0.12 | 0.09 | 90 | 0.10 | 0.10 | 74 | 0.35 | 0.15 | 40 |
| General | 0.08 | 0.18 | 25 | -0.05 | 0.09 | 90 | -0.13 | 0.11 | 76 | 0.03 | 0.12 | 38 |
| Musculoskeletal | -0.220 | 0.11 | 25 | 0.01 | 0.09 | 90 | 0.05 | 0.12 | 75 | 0.19 | 0.17 | 39 |
| Respiratory | 0.09 | 0.20 | 25 | -0.05 | 0.10 | 90 | -0.17 | 0.09 | 75 | -0.02 | 0.10 | 40 |
| Endocrine | 0 |  | 25 | 0.01 | 0.01 | 90 | 0.00 |  | 74 | 0.13 | 0.12 | 40 |
| Ocular | -0.06 | 0.04 | 24 | 0.12 | 0.11 | 87 | -0.06 | 0.02 | 75 | -0.06 | 0.03 | 40 |

**Table S2.** Matthews correlation coefficients (MCC) showing agreement between toxicities reported in humans and those reported in animal models

| Toxicity | Mice | | Rats | | Dogs | | Monkeys | |
| --- | --- | --- | --- | --- | --- | --- | --- | --- |
|  | MCC | No. of drugs | MCC | No. of drugs | MCC | No. of drugs | MCC | No. of drugs |
| Grades 3 and 4 |  |  |  |  |  |  |  |  |
| Hematologic | 0.62 | 28 | 0.20 | 90 | 0.26 | 76 | 0.43 | 40 |
| Cardiovascular | 0.37 | 27 | 0.091 | 90 | 0.07 | 77 | 0.19 | 40 |
| Gastrointestinal | 0.47 | 25 | 0.08 | 90 | -0.09 | 77 | 0.31 | 41 |
| Hepatic | 0.47 | 26 | 0.27 | 90 | 0.05 | 75 | 0.29 | 40 |
| Neurologic/ psychiatric | 0.05 | 24 | 0.19 | 90 | 0.11 | 75 | 0.23 | 40 |
| Cutaneous | 0.62 | 25 | 0.11 | 90 | 0.32 | 74 | 0.45 | 40 |
| Renal | 0.02 | 25 | -0.02 | 90 | 0.02 | 74 | -0.03 | 40 |
| Metabolic | 0.14 | 26 | 0.14 | 90 | 0.11 | 74 | 0.35 | 40 |
| General | 0.09 | 25 | -0.06 | 90 | -0.14 | 76 | 0.04 | 38 |
| Musculoskeletal | -0.28 | 25 | 0.01 | 90 | 0.05 | 75 | 0.21 | 39 |
| Respiratory | 0.09 | 25 | -0.05 | 90 | -0.17 | 75 | -0.04 | 40 |
| Endocrine | ^a^ | 25 | 0.08 | 90 | ^a^ | 74 | 0.26 | 40 |
| Ocular | -0.06 | 24 | 0.16 | 87 | -0.06 | 75 | -0.07 | 40 |
| All grades |  |  |  |  |  |  |  |  |
| Hematologic | 0.62 | 28 | 0.07 | 90 | 0.11 | 76 | 0.33 | 40 |
| Cardiovascular | 0.22 | 27 | 0.21 | 90 | 0.20 | 77 | 0.10 | 40 |
| Gastrointestinal | ^a^ | 25 | -0.086 | 90 | -0.04 | 77 | 0.20 | 41 |
| Hepatic | 0.36 | 26 | 0.16 | 90 | 0.03 | 75 | 0.11 | 40 |
| Neurologic/ psychiatric | 0.02 | 24 | 0.19 | 90 | 0.22 | 75 | 0.19 | 40 |
| Cutaneous | 0.32 | 25 | 0.30 | 90 | 0.20 | 74 | 0.32 | 40 |
| Renal | 0.12 | 25 | -0.06 | 90 | 0.00 | 74 | 0.09 | 40 |
| Metabolic | 0.32 | 26 | 0.12 | 90 | 0.27 | 74 | 0.30 | 40 |
| General | -0.011 | 25 | 0.006 | 90 | -0.05 | 76 | 0.09 | 38 |
| Musculoskeletal | 0.20 | 25 | -0.01 | 90 | 0.01 | 75 | 0.011 | 39 |
| Respiratory | 0.01 | 25 | -0.06 | 90 | -0.06 | 75 | -0.17 | 40 |
| Endocrine | ^a^ | 25 | 0.12 | 90 | -0.13 | =74 | 0.25 | 40 |
| Ocular | -0.09 | 24 | 0.11 | 87 | -0.08 | 75 | 0.12 | 40 |

^a^ Cannot be computed because at least one of the variables is constant.
